# Genomic characterization of colistin-resistant *Klebsiella pneumoniae* isolated from intensive care unit patients in Egypt

**DOI:** 10.1186/s12941-023-00632-9

**Published:** 2023-09-09

**Authors:** Eriny T. Attalla, Amal M. Khalil, Azza S. Zakaria, Dave J. Baker, Nelly M. Mohamed

**Affiliations:** 1 Microbiology and Immunology Department, Faculty of Pharmacy, Alexandria University, El-Khartoom Square, Azarita, Alexandria, Egypt; 2 Quadram Institute Bioscience, Norwich, UK

**Keywords:** *Klebsiella pneumoniae*, WGS, Colistin resistance, ICU, Egypt, *mgrB*, *mcr-1.1*, XDR, MPNP

## Abstract

**Background:**

Egypt has witnessed elevated incidence rates of multidrug-resistant *Klebsiella pneumonia*e infections in intensive care units (ICUs). The treatment of these infections is becoming more challenging whilst colistin-carbapenem-resistant *K. pneumoniae* is upsurging. Due to the insufficiently available data on the genomic features of colistin-resistant *K. pneumoniae* in Egypt, it was important to fill in the gap and explore the genomic characteristics, as well as the antimicrobial resistance, the virulence determinants, and the molecular mechanisms of colistin resistance in such a lethal pathogen.

**Methods:**

Seventeen colistin-resistant clinical *K. pneumoniae* isolates were collected from ICUs in Alexandria, Egypt in a 6-month period in 2020. Colistin resistance was phenotypically detected by modified rapid polymyxin Nordmann/Poirel and broth microdilution techniques. The isolates susceptibility to 20 antimicrobials was determined using Kirby-Bauer disk diffusion method. Whole genome sequencing and bioinformatic analysis were employed for exploring the virulome, resistome, and the genetic basis of colistin resistance mechanisms.

**Results:**

Out of the tested *K. pneumoniae* isolates, 82.35% were extensively drug-resistant and 17.65% were multidrug-resistant. Promising susceptibility levels towards tigecycline (88.24%) and doxycycline (52.94%) were detected. Population structure analysis revealed seven sequence types (ST) and K-types: ST383-K30, ST147-K64, ST17-K25, ST111-K63, ST11-K15, ST14-K2, and ST525-K45. Virulome analysis revealed yersiniabactin, aerobactin, and salmochelin siderophore systems in ˃ 50% of the population. Hypervirulence biomarkers, *iucA* (52.94%) and *rmpA/A2* (5.88%) were detected. Extended-spectrum β-lactamase- and carbapenemase-producers accounted for 94.12% of the population, with *bla*_CTX-M-15_, *bla*_NDM-5,_ and *bla*_OXA-48_ reaching 64.71%, 82.35%, and 82.35%, respectively. Chromosomal alterations in *mgrB* (82.35%) were the most prevailing colistin resistance-associated genetic change followed by deleterious mutations in ArnT (23.53%, L54H and G164S), PmrA (11.76%, G53V and D86E), PmrB (11.76%, T89P and T134P), PmrC (11.76%, S257L), PhoQ (5.88%, L322Q and Q435H), and ArnB (5.88%, G47D) along with the acquisition of *mcr-1.1* by a single isolate of ST525.

**Conclusions:**

In this study, we present the genotypic colistin resistance mechanisms in *K. pneumoniae* isolated in Egypt. More effective antibiotic stewardship protocols must be implemented by Egyptian health authorities to restrain this hazard and safeguard the future utility of colistin. This is the first characterization of a complete sequence of *mcr-1.1*-bearing IncHI2/IncHI2A plasmid recovered from *K. pneumoniae* clinical isolate belonging to the emerging high-risk clone ST525.

**Supplementary Information:**

The online version contains supplementary material available at 10.1186/s12941-023-00632-9.

## Background

*Klebsiella pneumoniae*, the most worrisome of the genus *Klebsiella*, accounts for about one-third of Gram-negative infections including hospital-acquired septicemia, pneumonia, meningitis, as well as surgical site, wound, and urinary tract infections [1]. These infections are life-threatening, affecting neonates and the elderly in intensive care units (ICUs) [1]. The problem is emphasized in the ICUs of lower middle-income countries where multidrug-resistant (MDR) *K. pneumoniae* is responsible for 15% of ICU-acquired infections [2]. As a part of this area, Egypt has witnessed elevated incidence rates of MDR *K. pneumoniae* recovered from patients admitted to ICUs reaching about 30% in 2021 [3]. The treatment of MDR *K. pneumoniae* infections is challenging and one of the treatment pillars for its eradication is carbapenem therapy [4]. As a consequence of the extensive usage of carbapenems, carbapenem-resistant *K. pneumoniae* (CRKP) began to upsurge more frequently in the past years, raising an alarm that effective treatment options are diminishing [3, 5]. CRKP emergence is linked to the global spread of carbapenemases, particularly, *K. pneumoniae* carbapenemase (KPC), New Delhi metallo-β-lactamase (NDM), and oxacillinase-48 (OXA-48) [6]. As a result, World Health Organization (WHO) published a list of antibiotic-resistant priority pathogens where carbapenem-resistant *Enterobacteriaceae* came at the first tier of the list, being identified as microorganisms of “critical priority” [7].

To fight against carbapenem resistance, colistin made a comeback to the forefront lines, becoming an antibiotic of last resort [8]. Colistin is a cationic polypeptide targeting mainly the negatively charged lipid A of the lipopolysaccharide (LPS) in the outer membrane of Gram-negative bacteria. Upon this electrostatic interaction, the bacterial membrane destabilizes, and the intracellular contents leak out causing bacterial death [9]. Unfortunately, colistin has witnessed rapid global resistance following its revival [8]. According to a systematic review conducted between 1987 and 2020, the universal incidence rate of colistin-resistant *K. pneumoniae* (ColRKp) was reported to be approximately 12%, comprising escalating continental trends reaching 3%, 10%, 16%, and 19%, in Africa, Asia, Europe, and America, respectively [10]. At the national level, the frequency of ColRKp reached 9.4% as reported by Zafer et al. conducting their study at the National Cancer Institute of Cairo University in 2019 [11]. It has been established that decreased susceptibility to colistin in *K. pneumoniae* is mainly attributed to mutations and genetic modifications in the chromosomally encoded genes, particularly, *mgrB*, *phoP*/*phoQ*, *pmrA*/*pmrB*, and *crrB*, and/or through the horizontal transfer of plasmid-mediated mobile colistin resistance (*mcr*) genes [12]. Among the ten different variants of *mcr* genes (*mcr-1* to *mcr-10*) reported so far, *mcr-1* remains the most dominant one universally [12, 13]. The worldwide dispersion of this gene in *K. pneumoniae* isolates has been facilitated by its carriage on a wide array of highly transferable plasmids of various incompatibility types, such as IncX4, IncI2, IncHI2, IncHII, IncFIIB, and IncP [12].

Both mutational and transferable mechanisms of colistin resistance result in a chemical alteration of lipid A molecule of the bacterial LPS via the addition of positively charged moieties, such as 4-amino-4-deoxy-l-arabinose (L-Ara4N) or phosphoethanolamine (pEtN), thus decreasing the lipid A negative charge and lowering the binding affinity of colistin to its target site [14, 15]. Nowadays, colistin is enlisted among the reserve group of antibiotics in the WHO list of essential medicines and its use is highly recommended to be tailored to specific clinical settings when no other treatment options are available [16].

In Egypt, no data was published on the molecular mechanisms of colistin resistance in ColRKp clinical isolates until 2018 [17]. To date, only a few studies had reported the occurrence of *mcr-1*, *mcr-2*, and mutated *mgrB* genes among the Egyptian isolates [11, 18–21]. In the current study, whole genome sequencing (WGS) approach was used to explore the genomic features of ColRKp isolates. Uncovering the resistome and virulome, as well as shedding light on the genetic basis of the mechanisms underlying colistin resistance will certainly provide a better understanding of the emergence and dissemination of this type of resistance in Egypt. Hence, the Egyptian health authorities will have the potential to develop more effective antibiotic stewardship protocols to restrain this hazard and safeguard the future utility of colistin. This is the first study presenting the detailed genomic characterization of ColRKp clinical isolates in Egypt and issuing a complete sequence of *mcr-1.1*-bearing IncHI2/IncHI2A plasmid recovered from a clinical isolate of *K. pneumoniae* belonging to the emerging high-risk clone ST525.

## Methods

### Collection and identification of clinical bacterial isolates

*Klebsiella* spp. isolates were obtained from a private hospital laboratory that has 8 satellite branches in Alexandria, Egypt, with a lab-to-lab patient service that almost covers the whole governorate of Alexandria. This laboratory receives about 400 to 500 *Klebsiella* spp. isolates every month. The criteria for collecting the isolates from this laboratory were set to be: “*Klebsiella* spp.”, “colistin resistance”, and “ICU patients”. Accordingly, a total of 17 colistin-resistant (Col-R) *Klebsiella* spp., numbered from K1 to K17, was obtained within a period of 6 months from July to December 2020. All isolates were preserved at − 20 °C in Luria-Bertani broth (LB, HiMedia, Mumbai, India) containing 20% glycerol and archived at − 80 °C. Before use, a fresh culture of each of the collected isolates was obtained by cultivation on MacConkey’s agar (HiMedia, Mumbai, India) and incubation for 24 h at 37 °C. The identification of the isolates was performed by Gram and capsule staining, followed by in-house prepared biochemical tests including indole production, methyl red, Voges-Proskauer, citrate utilization, triple-sugar iron, urease, and catalase tests. Further identification at the species level was performed using Vitek 2 compact system (bioMérieux, Marcy-L’Etoile, France) according to the manufacturer’s instructions.

### Modified rapid polymyxin Nordmann/Poirel test (MPNP)

In rapid polymyxin NP test (RPNP), the Col-R isolates metabolize the glucose present in the medium with the formation of acid that shifts the color of the phenol red pH indicator from orange to yellow, allowing the identification of these isolates through rapid visual observation [22]. The incorporation of EDTA in MPNP test increases the sensitivity of the technique enabling the detection of isolates harboring *mcr-1* gene [23]. Briefly, freshly grown colonies on LB agar (HiMedia Lab., Mumbai, India) were used to prepare a suspension with an optical density equivalent to 3.0–3.5 McFarland (≈ 10^9^ CFU/mL). A volume of 50 µL of this suspension was added to a well in a 96-well polystyrene plate containing 150 µL of one of the following solutions: RPNP solution (2.5% cation-adjusted Mueller–Hinton broth (CAMHB, HiMedia Laboratories, Mumbai, India); 0.005% phenol red (Sigma Chemical, St. Louis, USA); 1% D (+)-glucose anhydrous (Thermo Fisher Scientific, UK)), RPNP mixed with 5 µg/mL of colistin sulfate (Sigma Chemical, St. Louis, USA), RPNP supplemented with 80 µg/mL EDTA (Sigma Chemical, St. Louis, USA), and RPNP incorporating both colistin and EDTA [22, 23]. The plate was incubated at 37 °C unsealed and visually monitored during a period of 4 h for color change [22]. A solution of 0.85% NaCl was used as sterility control, while colistin-sensitive (Col-S) *E. coli* ATCC 8739 and Col-R *E. coli* EC13655 [24] were included as reference negative and positive controls, respectively.

### Antimicrobial susceptibility testing

The susceptibility of *K. pneumoniae* isolates to 20 antimicrobials was determined by Kirby-Bauer disk diffusion method. The disks for the following antibiotics were purchased from HiMedia Laboratories (Mumbai, India): piperacillin (PI), piperacillin/tazobactam (PIT), amoxicillin/clavulanate (AMC), ceftazidime (CAZ), ceftriaxone (CTR), cefepime (CPM), aztreonam (AT), imipenem (IPM), meropenem (MRP), ertapenem (ETP), amikacin (AK), gentamicin (GEN), tobramycin (TOB), azithromycin (AZ), doxycycline (DO), tigecycline (TGC), ciprofloxacin (CIP), and levofloxacin (LE). Whereas the disks for ceftazidime/avibactam (CZA) and sulfamethoxazole/trimethoprim (SXT) were obtained from Oxoid (Hampshire, UK). The test was carried out according to the Clinical Laboratory Standards Institute (CLSI, 2021) guidelines using Mueller–Hinton agar (HiMedia Laboratories, Mumbai, India) [25] and the results were interpreted in accordance with the breakpoints indicated in CLSI, except when using TGC disks, where the Food and Drug Administration (FDA) breakpoints for *Enterobacteriaceae* were adopted [26]. The broth microdilution technique (BMD) was performed in triplicate to determine the minimum inhibitory concentration (MIC) of colistin against the collected isolates using cation-adjusted Mueller–Hinton broth (CAMHB, HiMedia Laboratories, Mumbai, India), following the protocols recommended in M100-ED31 of the 2021 CLSI which considered isolates with a colistin MIC value of ≥ 4 µg/mL to be resistant. The quality control strain *E. coli* ATCC 25922 was included in the test.

### Whole genome sequencing (WGS)

WGS of the 17 tested isolates was carried out within the facility of the sequencing department at Quadram Research Institute, Norwich, UK. Bacterial DNA was extracted and purified using GeneJET Genomic DNA Purification Kit (Thermo Fisher Scientific, Vilnius, Lithuania) according to the manufacturer’s instructions. The extracted DNA was quantified using the Promega QuantiFluor® dsDNA System (Catalogue No. E2670), normalized to 5 ng/µL using PCR grade water, and run on a Promega GloMax® Discover Microplate Reader. Libraries were made using an Illumina 20-fold diluted DNA Prep reaction and amplified using custom 9 bp indexed primers. WGS was performed on an Illumina NextSeq 500 instrument using a High Output Flowcell NextSeq 500/550 High Output Kit v2.5 (300 Cycles, Illumina Catalogue 20024908) according to the Illumina denaturation and loading recommendations which included a 1% PhiX spike (PhiX Control v3 Illumina Catalogue FC-110-3001). The libraries were quantified using the GloMax and QuantiFluor® dsDNA kit. Libraries were pooled following quantification and the final pool was double-SPRI size selected between 0.5 and 0.7× bead volume using the beads supplied in the Illumina® DNA Prep kit. The final pool was quantified on a Qubit 3.0 instrument and run on a D5000 Screentape (Agilent Catalogue No. 5067-5579) using the Agilent tapestation4200 to calculate the final library pool molarity. The generated BCL files were converted to FASTQ format using bcl2fastq v2.20.0.422 software [27] for downstream analysis.

### Bioinformatic analysis

The FASTQ files were quality-checked and processed using fastp v0.23 [28] where low quality nucleotides of Q-score < 20 were trimmed. Shovill v1.1.0 (https://github.com/tseemann/shovill) was used for de novo assembly and the quality was evaluated using QUAST v5.0.2 [29] for genome contiguity and BUSCO v5.2.2 [30] for genome completeness. The obtained contigs were queried utilizing the pipelines available on Center for Genomic Epidemiology (CGE) (https://www.genomicepidemiology.org/) (accessed on 31 May 2022) to perform multilocus sequencing typing (MLST v2.0) and to identify acquired antimicrobial resistance (AMR) genes (ResFinder v4.1). Chromosomal point mutations for cephalosporins, fluoroquinolones, and carbapenems resistance were identified (PointFinder v4.1) and plasmid incompatibility (Inc) groups were detected using PlasmidFinder v2.1. The Institut Pasteur website (https://bigsdb.pasteur.fr/klebsiella/) (accessed on 15 June 2022) was used for typing of *wzi* and *wzc* alleles and locating heavy metal resistance genes. Virulence genes were detected using the Institut Pasteur website and Virulence Factor Database (VFDB) [31]. Kaptive database was used for capsular (K) and lipopolysaccharide (O-antigen) locus typing (https://kaptive-web.erc.monash.edu/) (accessed on 20 June 2022). The presence of *mcr* variants was investigated using ResFinder v4.1. Chromosomal loci disruptions or alterations linked to colistin resistance (e.g., alterations in *mgrB* locus (gene and its promoter), the genetic environment of *mgrB* including *kdgR*, *yobH*, *yebO*, *yobF*, and *cspC*, *phoPQ*, *pmrAB*, *pmrC, pmrD*, *crrB, arnBCADTEF operon,* and *ramA*) were explored *in silico* using the nucleotide basic local alignment search (BLASTn) tool of National Center of Biotechnology Information (NCBI) (https://blast.ncbi.nlm.nih.gov/Blast.cgi) by aligning the assembled contigs against the wild type gene sequence of *K. pneumoniae* subsp. *pneumoniae* HS11286 (GenBank accession number: NC_016845.1). Any change in the protein level was analyzed using the NCBI BLASTx tool (https://blast.ncbi.nlm.nih.gov/Blast.cgi?PROGRAM=blastx&PAGE_TYPE=BlastSearch&LINK_LOC=blasthome) followed by the Protein Variation Effect Analyzer tool (PROVEAN) (https://provean.jcvi.org/index.php) (accessed on 6 June 2022) to predict the amino acids substitution/deletion effect on the functional biological activity of the protein. ISfinder was used to look for insertion sequences [32]. Finally, phylogenetic relatedness between isolates was investigated using CSI phylogeny [33] by setting NC_009648.1 as the reference genome. The phylogenetic tree was visualized using Interactive Tree of Life V6.5 (iTOL) [34].

### Construction of *mcr-1.1*-bearing plasmid

PlasmidSPAdes v3.15.3 [35] was used to generate the plasmid contigs in sample K2. The node harboring *mcr-1.1* was queried against *K. pneumoniae* (taxid:573) using BLASTn tool, followed by mapping all the generated plasmid contigs from the tested sample against the available plasmid sequences online. The complete sequence of the constructed plasmid pEGY_KP9814_MCR1 was generated by assembling multiple contigs while overlapping sequences were manually curated. The plasmid annotation was performed using the NCBI prokaryotic genome annotation pipeline (PGAP) [36]. Plasmid Inc groups and AMR genes were detected using PlasmidFinder v2.1 [37] and ResFinder v4.1 [38] tools, respectively. The circular comparison map between pEGY_KP9814_MCR1 and other similar plasmids was generated using CGview server v1.1.2 (http://stothard.afns.ualberta.ca/cgview_server/) (accessed on 1 April 2023) after mining similar plasmid sequences from NCBI. Comparative analysis of *mcr-1.1* genetic environment was performed using Clinker [39].

## Results

### Clinical characteristics of the collected isolates

The collected isolates were preliminary identified as *Klebsiella* spp. by conventional methods (Additional file 1: Figs. S1 and S2) and their identities were further confirmed to be *K. pneumoniae* subspecies *pneumoniae* using Vitek 2 compact system. The clinical origin of these isolates was as follows: blood (n = 8), mini-bronchoalveolar lavage (mini-BAL, n = 3), tracheal aspirate (n = 2), urine (n = 2), swab (n = 1), and sputum (n = 1). Most of the isolates were recovered from geriatrics (n = 10) with a mean age of 67 years old, followed by neonates (n = 6) with an age range of 4–15 days, while a single isolate was obtained from a 32-year-old male. The detailed demographic data including clinical origin, age, gender, patient code, and date of collection are provided in Additional file 2: Table S1.

### Phenotypic detection of colistin resistance

The MPNP test was performed for the initial screening of colistin resistance and the presumptive detection of *mcr-1* gene. All isolates grew in the presence of colistin- and colistin/EDTA-containing RPNP solution, indicating colistin resistance and a presumptive absence of *mcr-1*, respectively. The growth of the tested isolates was evidenced by the color change of the pH indicator from orange to yellow (Additional file 1: Fig. S3). Colistin resistance was confirmed by BMD where isolates displayed colistin MIC values ranging from 8 to 128 µg/mL (Additional file 2: Table S2).

### Genomic features of ColRKp isolates

**Fig. 1:**
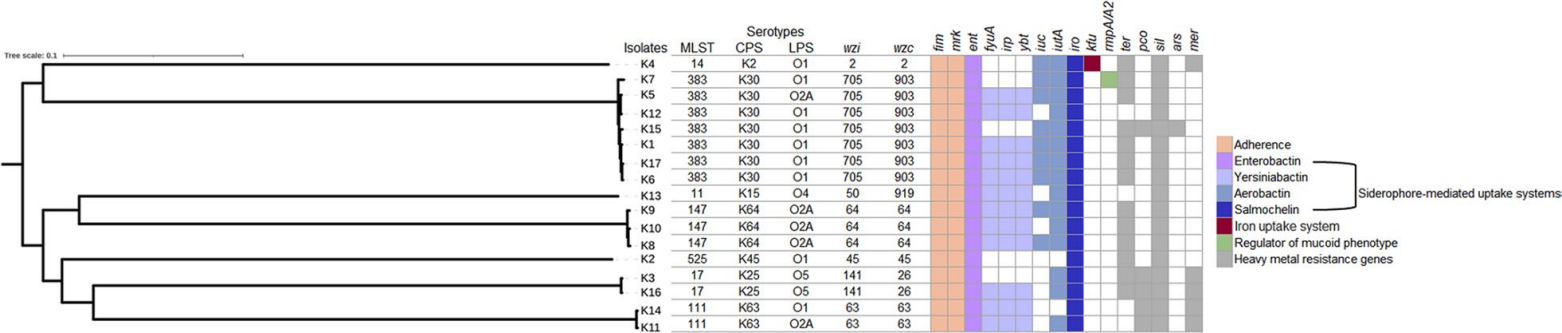
Illustration of the multilocus sequence typing (MLST), capsule polysaccharide (CPS), lipopolysaccharide (LPS), *wzi* and *wzc* gene alleles, and a heatmap of virulence determinants and heavy metal resistance genes of colistin-resistant *K. pneumoniae* isolates. On the left, the phylogenetic tree is rooted at the mid-point showing clustering of the isolates by sequence types and visualized by iTOL tool. The virulence pattern is portrayed as gene present (colored) or absent (white) for the following virulence determinants: adherence (light orange), enterobactin (lavender), yersiniabactin (light blue), aerobactin (mid-tone blue), salmochelin (blue), iron uptake system by *kfu* (dark red), and regulator of mucoid phenotype by *rmpA*/*rmpA2* (light green). Heavy metal resistance genes coding for tellurium (*ter*), copper (*pco*), silver (*sil*), arsenic (*ars*), and mercury (*mer*) resistance are denoted as gene present (grey) or absent (white)

WGS using Illumina short read sequencing technology was performed for the 17 ColRKp isolates. De novo assembly generated draft genomes with a size range of 5.4 to 6 Mb. The average number of contigs per isolate was 245 with an N_50_ of 114,109 bp and mean G+C content of 56.82%. Assembly statistics are provided in Additional file 2: Table S3. The population structure of *K. pneumoniae* strains was investigated using the genome sequence data and their phylogenetic relatedness is represented in Fig. 1 where each well-defined branch in the phylogenetic tree comprises strains of the same sequence types (ST) clustering together. Seven distinct STs featured the studied collection according to the MLST allelic analysis, out of which four STs were represented by more than one isolate: ST383 (7/17), ST147 (3/17), ST17 (2/17), and ST111(2/17), while ST11, ST14, and ST525 were displayed each by a single isolate. Kaptive database was used for capsular polysaccharide (K-type) and lipopolysaccharide (O-antigen) characterization. It predicted 7 different K-types: K-2, K-15, K-25, K-30, K-45, K-63, and K-64 which were found to be correlated with ST14, ST11, ST17, ST383, ST525, ST111, and ST147 lineages, respectively. The association of ST383 with K-30 capsular type appears to be the most ubiquitous in the collection. O-serotyping revealed 4 different O antigens: O1, O2A, O4, and O5, with O1 and O2A serotypes being the most abundant, accounting for 52.94% and 29.41% of the isolates, respectively. Each K-type, *wzi*, and *wzc* alleles were found to belong to the same ancestry lineages. Meanwhile, O-serotype was associated with variable STs (Fig. 1).

Virulence profiles of ColRKp isolates were investigated to infer to the isolates likelihood of causing severe infections. All isolates harbored the core pathogenicity factors, *fim* and *mrk* genes cluster, coding type 1 and type 3 fimbriae which are involved in the adherence and biofilm formation, respectively, as well as genes encoding the core siderophore enterobactin, *entABCDEFS*, responsible for iron scavenging from host cells. Additional acquired siderophore systems which contribute to *K. pneumoniae* virulence were detected in more than half of the population, namely: yersiniabactin (synthesized by *ybtAEPQSTUX*, regulated by *irp1/2* and its receptor encoded by *fyuA*), aerobactin (encoded by *iucABCD* and transported into bacterial cells by its corresponding receptor encoded by *iutA*), and salmochelin (encoded by *iro* genes). None of the isolates harbored *iroBCD* genes, whereas both *iroN* and *iroE* were found in all of the isolates.

Noteworthy, yersinia high-pathogenicity island (HPI) which comprises *ybtAEPQSTUX, irp1, irp2*, and *fyuA* [40] was detected in 11 isolates: K5, K6, K8, K9, K10, K11, K12, K13, K14, K16, and K17. The presence of this HPI enables the producing isolates of enhanced iron sequestration and bacterial proliferation. The biomarker of hypervirulence, *iucA*, was detected in 9 isolates while *rmpA/A2*, regulator of mucoid phenotype, was found in a single isolate (Fig. 1). An additional pathway for iron transportation, *kfu* (*Klebsiella* ferric uptake) system, was found in K4 isolate. Additional virulence determinants including mucoviscosity-associated gene A (*magA*) and allantoin utilization genes (*allS* and *allABCDR*) were investigated using BLASTn tool and VFDB, respectively, however, they were not detected in any of the studied isolates.

Multiple heavy metal resistance genes coding for tellurium (*ter*), copper (*pco*), silver (*sil*), arsenic (*ars*), and mercury (*mer*) resistance were detected among the isolates with *silR* being present in all isolates and *ars* being exclusively found in K15 isolate. The distribution of virulence and heavy metal resistance genes among the isolates is detailed in Additional file 2: Table S4.

**Fig. 2:**
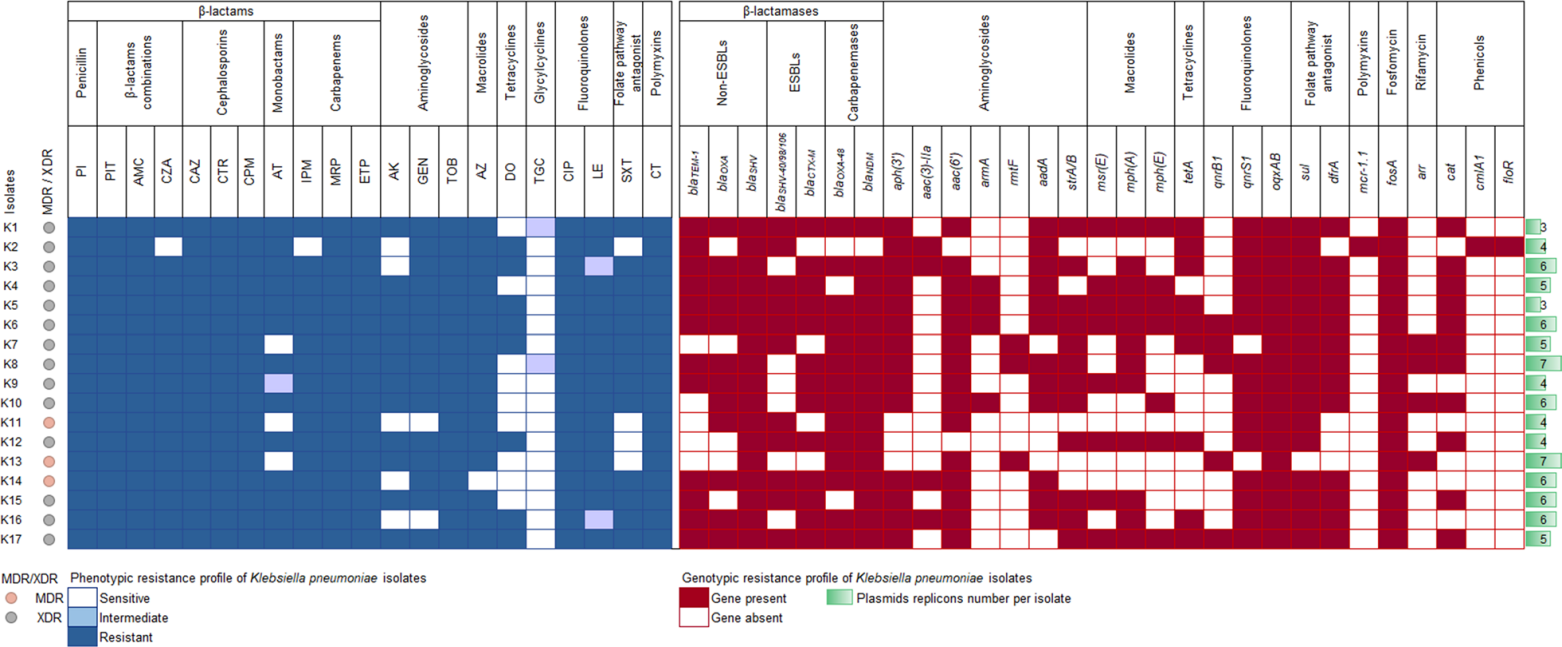
Heatmap of the phenotypic and genotypic antimicrobial resistance profile and plasmid replicon numbers of colistin-resistant *K. pneumoniae* isolates. The phenotypic profile is mapped by white, light blue, and blue colors which respectively correspond to the isolate sensitivity, intermediate susceptibility, and resistance to the antibiotic specified in the column header. The genotypic profile is denoted by dark red or white colors indicating the presence or absence of the corresponding gene, respectively. The green bars on the right illustrate the number of plasmid replicons detected per isolate. The circle symbols on the left indicate the resistance status of the isolates as multidrug-resistant (MDR, pink) or extensively drug-resistant (XDR, grey). *PI* piperacillin, *PIT* piperacillin/tazobactam, *AMC* amoxicillin/clavulanate, *CZA* ceftazidime/avibactam, *CAZ* ceftazidime, *CTR* ceftriaxone, *CPM* cefepime, *AT* aztreonam, *IPM* imipenem, *MRP* meropenem, *ETP* ertapenem, *AK* amikacin, *GEN* gentamicin, *TOB* tobramycin, *AZ* azithromycin, *DO* doxycycline, *TGC* tigecycline, *CIP* ciprofloxacin, *LE* levofloxacin, *SXT* sulfamethoxazole/trimethoprim, *CT* colistin

PlasmidFinder identified a total of 17 distinct plasmid replicon types with a minimum of 3 and a maximum of 7 in each of the isolates (Fig. 2). The most prevalent plasmid replicon type belonged to the Inc family, dominated by IncL and IncFIB(pQil) which were detected in 76.47% and 64.71% of the isolates, respectively. These were followed by IncFII_K_, IncFIB(pNDM-Mar), and IncHI1B(pNDM-MAR) which prevailed in 58.82% of the investigated collection, with the latter two Incs co-existing together. Other Inc types were identified including IncFII, IncFIB(pKPHS1), IncFIB_K_, IncFIB_K_(pCAV1099-114), IncHI2/2A, and IncX3/M1/R. Three types of Col plasmid replicons were observed including ColRNAI (n = 4; 23.53%), Col440II (n = 3; 17.65%), and Col440I (n = 1; 5.88%). The plasmid replicon types for each isolate are listed in Additional file 2: Table S4.

### Phenotypic antimicrobial resistance profile

The Kirby–Bauer disk diffusion method was employed to assess the susceptibility of ColRKp isolates to a panel of 20 antibiotics which were selected based on the CLSI guidelines provided for *K. pneumoniae* infections. More than half of the tested antibiotics (11/20) were ineffective against all the tested isolates, namely: PI, PIT, AMC, CTR, CAZ, CPM, MRP, ETP, TOB, CIP, and LE. A percentage of 94.12% of the isolates were resistant to CZA, IPM, and AZ. Moreover, high levels of resistance, exceeding 70%, were detected for AT, AK, GEN, and SXT. While most of the isolates showed alarming resistance levels to 18/20 of the evaluated antimicrobials, TGC and DO retained their efficacy against 88.24% and 52.94% of the studied isolates, respectively (Fig. 2). The tested *K. pneumoniae* isolates were predominately extensively drug-resistant (XDR) by being non-susceptible to at least one antibiotic in all but one or two investigated antimicrobial categories. Meanwhile, a small proportion (17.65%) of the isolates exhibited an MDR phenotype by displaying resistance to at least one agent in ≥ 3 antimicrobial classes (Fig. 2).

### Genotypic analysis of antimicrobial resistance determinants

ResFinder identified multiple acquired AMR genes (Fig. 2) which are in line with the XDR and MDR phenotypes displayed by the isolates. All isolates were extended spectrum β-lactamase (ESBL) producers except for K13. The ESBL-encoding genes detected among the studied population were highly diverse comprising *bla*_SHV-40_, *bla*_SHV-98_, *bla*_SHV-106_, *bla*_CTX-M-14b_, *bla*_CTX-M-15_, *bla*_CTX-M-163_, *bla*_CTX-M-194_, and *bla*_CTX-M-219_, with *bla*_CTX-M-15_ and *bla*_CTX-M-14b_ being the most frequently observed in 64.71% and 52.94% of the isolates, respectively. However, *bla*_SHV-106,_*bla*_CTX-M-163_, *bla*_CTX-M-194_, and *bla*_CTX-M-219_ were less common, being found in 5.88% of the isolates (Additional file 2: Table S4). Among carbapenemases, *bla*_NDM_ genes were the most frequently encountered (n = 16), with *bla*_NDM-5_ variant being the most common among the isolates (n = 14) as well as *bla*_OXA-48_ with an equal prevalence, whereas *bla*_KPC_, *bla*_VIM_, and *bla*_IMP_ were absent among the studied isolates. Additional β-lactamases including non-ESBLs were detected (Fig. 2) and their corresponding variants are listed in Additional file 2: Table S4. PointFinder analysis of *ompK35*, *ompK36*, and *ompk37* genes encoding outer membrane proteins revealed multiple chromosomal mutations that have an impact in reducing membrane permeability (Additional file 2: Table S4). Thus, explaining the observed phenotypic resistance to carbapenems and cephalosporins for those isolates lacking carbapenemases or ESBL-encoding genes. Fifteen genes accountable for aminoglycosides resistance were recognized: *aph(3′)-Ia, aph(3′)-VI, aph(3′)-VIb, aac(3)-IIa, aac(6′)-Il, aac(6′)-Ib, aac(6′)-Ib-Hangzhou, aac(6′)Ib-cr, armA, rmtF, aadA1, aadA2, aadA2b, strA*, and *strB* (Additional file 2: Table S4). Among these genes, *armA* and *rmtF* which are associated with pan-aminoglycoside resistance were present in 7 isolates. The aminoglycoside resistance gene, *aph(3′)-Ia*, was the most abundant (n = 14) followed by *aac(6′)Ib-cr* (n = 13) which additionally confers resistance to ciprofloxacin (Additional file 2: Table S4). Moreover, fluoroquinolones resistance was mediated by three mechanisms: *qnrS1* and *qnrB1* plasmid-mediated genes, *oqxAB* chromosomally encoded efflux pump, and the chromosomal loci mutations detected in *gyrA*, *parC*, and *acrR* (Additional file 2: Table S4). Doxycycline resistance was mainly attributed to the acquisition of *tetA* gene while other variants of *tet* genes were completely absent.

Different variants of *sul* and *dfrA* genes (Additional file 2: Table S5) co-existed in 76.47% of the isolates mediating resistance to sulfamethoxazole and trimethoprim, respectively. A comparable distribution of *sul1* and *sul2* genes was observed among the isolates, with *sul1* being detected in 76.47% and *sul2* in 70.59% of the isolates. Additionally, the co-occurrence of both genes was spotted in 58.82% of the bacterial collection. Conversely, *sul3* was less frequent, being detected in a single isolate, K2. Chloramphenicol resistance was primarily mediated by chloramphenicol acetyltransferases, *catA1* and *catB3*, detected in 64.71% of isolates, whereas chloramphenicol efflux pumps, *floR* and *cmlA*, were carried by a single isolate, K2. Additional resistance determinants were detected for various classes of antimicrobials such as *msr(E)*, *mph(A)*, and *mph(E)* mediating resistance to macrolides; *fosA* encoding fosfomycin resistance and *arr-2* and *arr-3* coding for rifamycin resistance.

### Molecular characterization of colistin resistance mechanisms

**Fig. 3:**
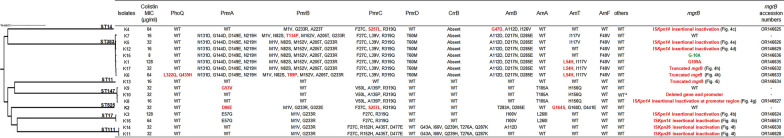
Colistin MIC and sequence analysis of colistin resistance genes/proteins in the tested *K. pneumoniae* isolates compared to the wild-type sequence of *K. pneumoniae* subsp. *pneumoniae* HS11286 (GenBank accession number: NC_016845.1). Mutations, deleterious amino acid substitutions, insertional inactivation, or deletion potentially associated with colistin resistance are shown in red bold format. Deleterious and neutral amino acid substitutions were evaluated by PROVEAN tool. Green bold mutation in *mgrB* upstream region at position − 10, when referring to the first nucleotide upstream *mgrB* start codon as − 1, is suspected to be associated with colistin resistance. Others refer to PhoP, ArnC, ArnD, ArnE, RamA, *kdgR*, *yobH*, *yebO*, *yobF*, and *cspC* with ∆ indicating partial deletion of *yobH* and complete deletion of *yebO*, *yobF*, and *cspC*. On the left, isolates are clustering based on their sequence types that are indicated above each branch of the phylogenetic tree. Isolate shown in bold format (K2) harbored plasmid-mediated *mcr-1.1* gene. *WT* wild-type

**Fig. 4:**
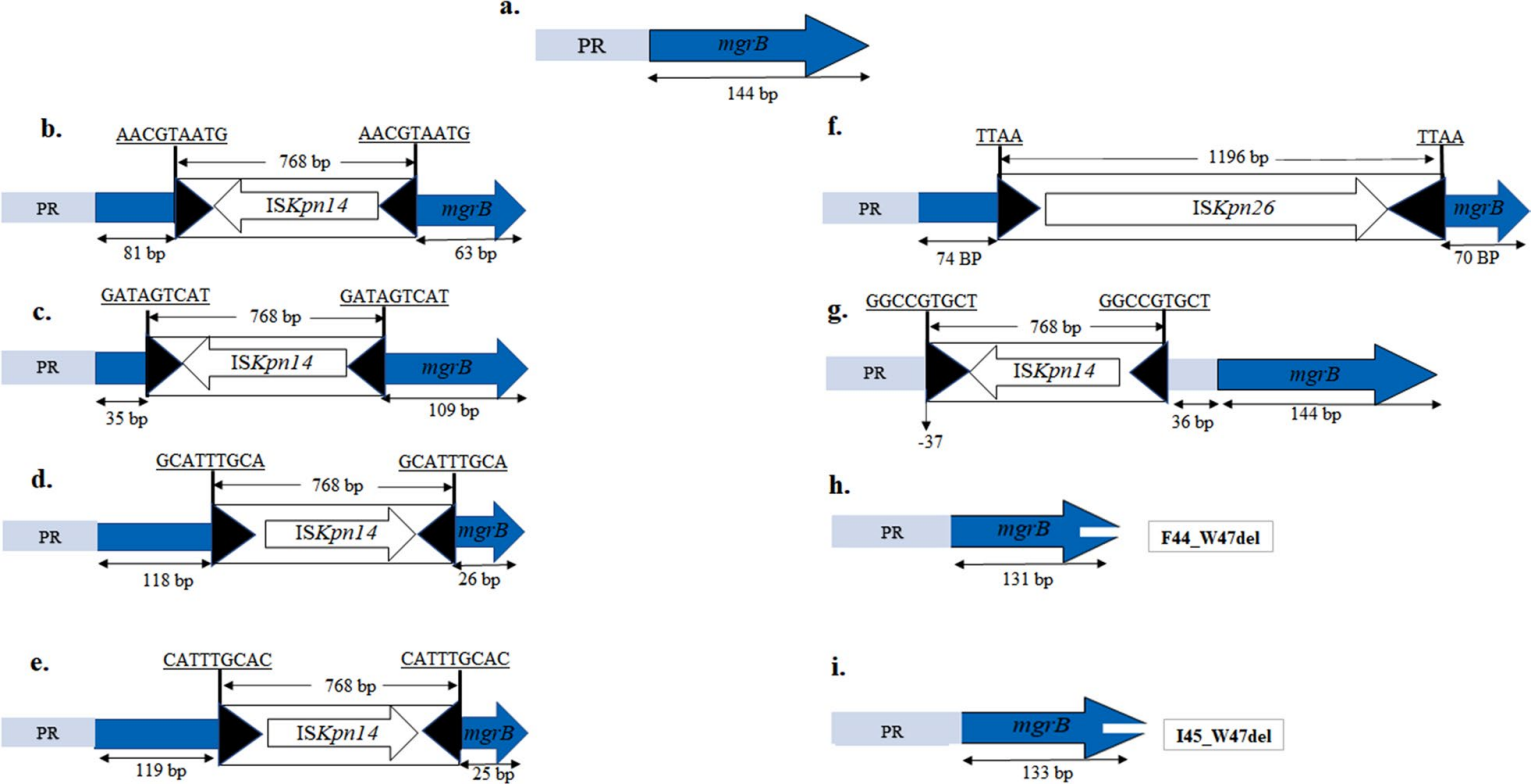
Schematic representation of the insertional inactivation disrupting *mgrB* locus along with an illustration of truncated *mgrB* gene. **a** Intact wild-type *mgrB* locus of *K. pneumoniae* HS11286 showing *mgrB* gene (blue arrow) and promoter region (PR) (light blue rectangle); **b** *mgrB* gene inactivated by IS*Kpn14* at nucleotide position + 81, **c** at nucleotide position + 35, **d** at nucleotide position + 118, and **e** at nucleotide position + 119; **f** *mgrB* gene disrupted by IS*Kpn26* at nucleotide + 74; **g** *mgrB* promoter region interrupted by IS*Kpn14* at nucleotide position − 37 when referring to the first nucleotide upstream *mgrB* start codon as − 1. Target sites duplication of 4 and 9 bp are underlined, while the black triangles represent the left and right inverted repeats of the insertion sequences. **h** Truncated *mgrB* showing the deletion of the terminal 13 nucleotides yielding a MgrB of 43 amino acids instead of 47, F44_W47del indicates the deletion of amino acids from position 44 (phenylalanine) to position 47 (tryptophan); **i** truncated *mgrB* of 133 bp sequence producing a MgrB of 44 amino acids, I45_W47del indicates the deletion of amino acids from position 45 (isoleucine) to position 47 (tryptophan). The diagram is not made to scale

Screening for plasmid-mediated *mcr* genes by ResFinder revealed that a single isolate, K2 belonging to ST525, harbored *mcr-1.1*, while other *mcr* variants were completely absent among the investigated collection. Furthermore, the *mcr*-bearing isolate had additional chromosomal mutations in *pmrA, pmrC*, and *arnT* leading to D86E, S257L, and G164S deleterious substitutions, respectively, as predicted by PROVEAN tool. Inspection of further chromosomal mutations conferring colistin resistance showed deleterious substitutions within PhoQ (L322Q and Q435H in K6 isolate), PmrA (G53V in K9 isolate), PmrB (T134P in K7 isolate and T89P in K6 isolate), PmrC (S257L in K4 isolate), ArnB (G47D in K4), and ArnT (L54H in K1, K6 and K17 isolates). Relatively conserved PmrD, ArnA, and ArnF as well as wild-type PhoP, ArnC, ArnD, ArnE, RamA, KdgR, YobH, YebO, YobF, and CspC were detected in all isolates except K10 isolate which lacked the latter four proteins (Fig. 3). It was noted that isolates that were clustered to a specific ST possessed similar neutral amino acids substitution within PmrAB, PmrC, PmrD, ArnA, ArnF, and CrrB (Fig. 3). The *crrB* gene could not be detected in isolates belonging to the clonal groups ST383 and ST14. Alteration in *mgrB* locus appears to be the predominant mechanism mediating colistin resistance where it was encountered in 82.35% of the tested collection. Six isolates belonging to ST14, ST383, and ST17 had an IS*Kpn14* (member of IS1 family) insertional inactivation targeting different nucleotide positions in *mgrB* (Figs. 3 and 4b–e), while an insertional inactivation of the *mgrB* promoter by IS*Kpn14* was observed in K8 isolate (Figs. 3 and 4g). The disruption of *mgrB* at nucleotide position + 74 by a member of IS5 family, IS*Kpn26*, was detected in two isolates of ST111 (Figs. 3 and 4f). Other alterations in *mgrB* included partial deletion of *mgrB* leading to the generation of truncated *mgrB* gene and an incomplete MgrB protein in three isolates (Figs. 3 and 4h, i), complete deletion of *mgrB* locus with the neighboring genes being completely (*yebO*, *yobF*, and *cspC*) and partially (*yobH*) deleted in K10 isolate, G109A *mgrB* chromosomal mutation leading to G37S deleterious amino acid substitution in K1 isolate, and a chromosomal mutation of guanine into adenine in the upstream region of *mgrB*, particularly at nucleotide position − 10, when referring to the first nucleotide upstream *mgrB* start codon as − 1 in K15 isolate. Collectively, our results indicate that genetic alterations associated with colistin resistance are diverse among the Egyptian strains, with K2 isolate being featured among the collection for having dual chromosomal and plasmid-mediated mechanisms of colistin resistance.

Of note, 88.24% of the isolates were colistin-resistant but TGC-sensitive, making this observation interesting for the investigation of additional genes related to colistin/TGC cross-resistance including the efflux pump mechanisms *acrAB* and *soxSR*. Wild-type gene and protein sequences were revealed across all isolates through comparative analysis against the reference sequence of *K. pneumoniae* HS11286.

### Characterization of pEGY_KP9814_MCR1 plasmid and its comparison to similar plasmids

**Fig. 5:**
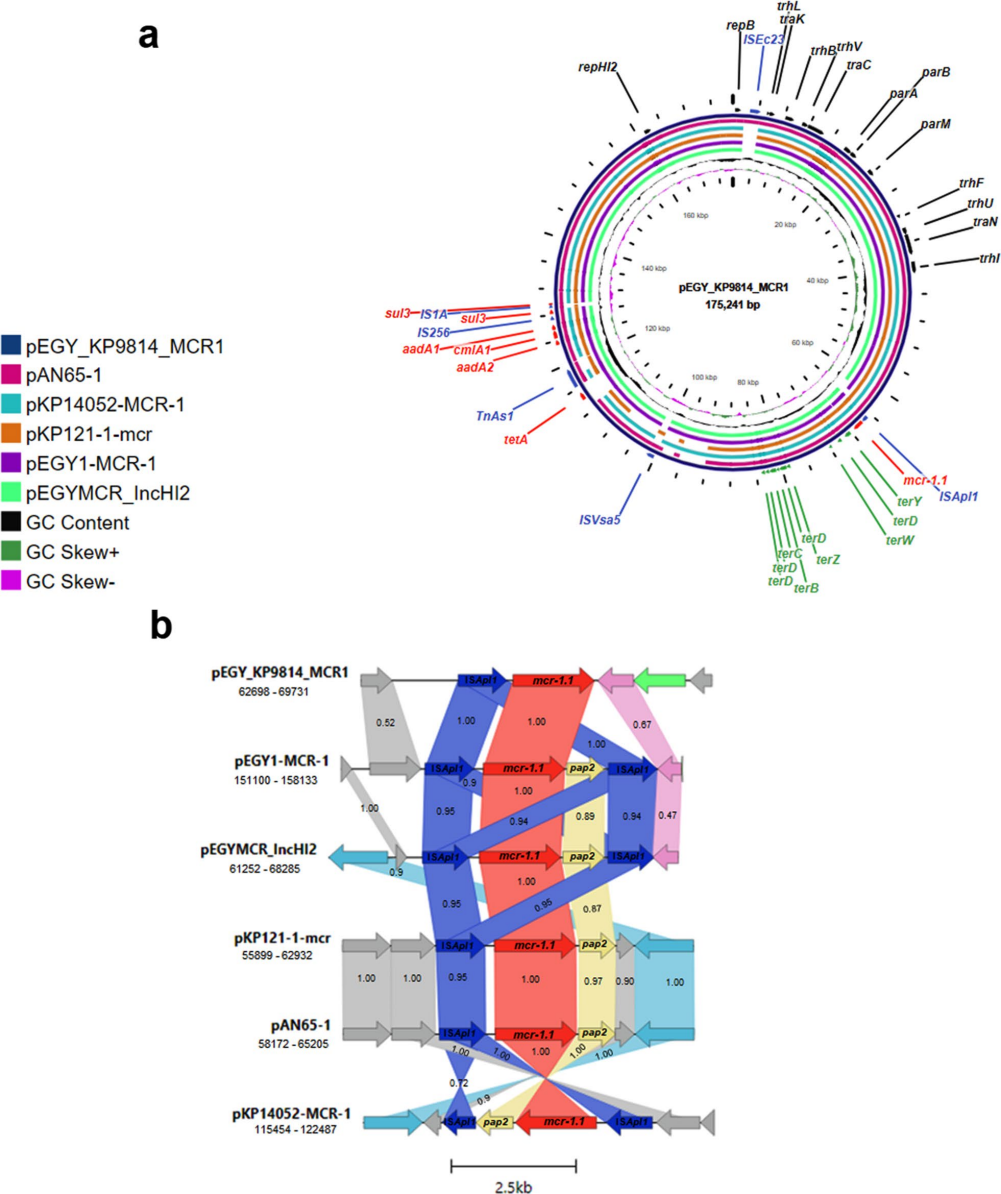
**a** CGview comparison of pEGY_KP9814_MCR1 with similar IncHI2 *mcr-1.1*-bearing plasmids retrieved from NCBI database. Circles from outside to inside correspond to the coding sequence regions of pEGY_KP9814_MCR1 (dark blue) with its size indicated in the middle of the circles, pAN65-1 (pink), pKP14052-MCR-1 (turquoise), pKP121-1-mcr (orange), pEGY1-MCR-1 (purple), and pEGYMCR_IncHI2 (spring green). The labels in the outermost circle represent the annotation of genes related to antibiotic resistance (red), tellurium resistance (green), transposases (blue), and plasmid replication, transfer, and segregation (black). Genomic regions covered by BLASTn are represented by a solid color whereas white gaps indicate regions not covered by BLASTn; **b** schematic illustration of the genetic environment surrounding *mcr-1.1* gene in pEGY_KP9814_MCR1 compared to previously reported plasmids recovered from *K. pneumoniae* and *E. coli*. The colored shading between genetic loci indicates homology with a percentage identity demonstrated within the shaded region. Red, blue, yellow, pink, light blue, green, and grey arrows represent open reading frames corresponding to *mcr-1.1*, IS*Apl1*, *pap2*, PP2C-encoding gene, tyrosine-type recombinase/integrase, *terY*, and different hypothetical proteins, respectively. The figure was generated using Clinker software

The *mcr-1.1-*bearing plasmid was harbored by a *K. pneumoniae* ST525 clinical isolate obtained from a tracheal aspirate of a male patient. The plasmid, designated pEGY_KP9814_MCR1 (GenBank ID OQ215737I), was 175,241 bp long with an IncHI2 and IncHI2A backbone. It contained 174 CDS and had an average G+C content of 46%. The plasmid harbored essential proteins responsible for plasmid replication (Rep), conjugative transfer (Tra and Trh), maintenance, and segregation (Par). Co-occurring AMR genes were identified alongside *mcr-1.1* such as *aadA1, aadA2b, tetA, cmlA1*, and *sul3*, whereas *sul3* was interrupted by IS1-like element IS1A family transposase. Furthermore, tellurium resistance genes (*terBCDWYZ*) and genes coding transposases were carried by the plasmid. Comparative analysis of pEGY_KP9814_MCR1 against similar published plasmids using BLASTn revealed high sequence identity with IncHI2 *mcr-1*-positive plasmids recovered from *E. coli* in Egypt namely pEGY1-MCR-1 of raw milk cheese origin (GenBank ID CP023143.1) and pEGYMCR_IncHI2 obtained from chicken carcass (GenBank ID MT499884.1), both exhibiting 99.99% sequence identity and query coverage of 96% and 95%, respectively. Moreover, pEGY_KP9814_MCR1 shared a significant sequence similarity to three IncHI2 plasmids of clinical origin identified in *K. pneumoniae* in Asia; pAN65-1 (GenBank ID MK355502.1) and pKP121-1-mcr (GenBank ID NZ_CP031850.1) in China, as well as pKP14052-MCR-1 (GenBank ID MH715960.1) in Taiwan, all showing 99.73% nucleotide identity and 93%, 93%, and 88% sequence length, respectively (Fig. 5a).

### Genetic context of *mcr-1.1*

In silico analysis of the genetic environment of *mcr-1.1* in pEGY_KP9814_MCR1 revealed a sole IS*Apl1* insertion sequence in the upstream region of *mcr-1.1*. A single upstream copy of IS*Apl1* was identified as well in pAN65-1 and pKP121-1-mcr, however, a composite transposon of IS*Apl1*-*mcr-1.1*-orf-IS*Apl1* was carried by pEGYMCR_IncHI2, pEGY1-MCR-1, and pKP14052-MCR-1. Furthermore, the gene encoding phosphatase protein (PAP2) was located directly after *mcr-1.1* in the previously published plasmids. Whereas pEGY_KP9814_MCR1 was distinguished by carrying a protein phosphatase (PP2C) at the downstream region of *mcr-1.1* followed by a tellurium resistance gene (*terY*) (Fig. 5b). Marked similarity (100% nucleotide identity and 57% query coverage) was observed in the flanking regions surrounding *mcr-1.1* in pEGY_KP9814_MCR1 and pEGY1-MCR-1, with the latter plasmid carrying similar hypothetical protein as in pEGY_KP9814_MCR1 and a partial sequence of PP2C. Comparing the genetic context of the remaining four plasmids with pEGY_KP9814_MCR1 revealed 99.89–100% nucleotide identity with varying query coverage (Fig. 5b).

## Discussion

The global crisis of AMR had escalated during the last decade with the emergence of bacterial resistance to last resort antibiotics such as colistin, especially when it is encountered in a clinically problematic pathogen such as *K. pneumoniae* [1, 8]. In the presented work, we investigated the genomic features of ColRKp collected from adult and neonatal ICUs in Alexandria, Egypt, in a 6-month period. The clinical origin of 47% of the isolates was from blood cultures, similar to a study conducted at the National Cancer Institute of Cairo University [11] which signifies a worrying scenario due to the high risk of mortality caused by bloodstream infections, especially in case of colistin and carbapenem resistance [41].

The performed MPNP test detected colistin resistance in the 17 collected isolates and the results were confirmed by the recommended BMD technique, however, MPNP test failed to detect *mcr-1.1* harbored by K2 isolate, which was identified subsequently by WGS. This could be justified by the presence of additional chromosomal mutations in this isolate impeding the EDTA-mediated inhibitory effect. Therefore, the growth of isolates in the presence of colistin/EDTA-containing RPNP solution might indicate the absence of *mcr* or the presence of *mcr* along with chromosomal mutations related to colistin resistance creating a false negative result similar to the one obtained in the current study. In addition, the sensitivity of this phenotypic test is indeed not comparable to the molecular detection methods of colistin resistance. Therefore, a cautious interpretation of the MPNP results is recommended along with cross-checking by molecular techniques for reliability.

The genomic analysis of the ColRKp isolates revealed a highly divergent genetic makeup comprising multiple STs, K- and O-types, thus, highlighting the genomic plasticity of the strains circulating in Egypt. ST383, an epidemic high-risk clone previously reported in ColRKp isolates in Greece [42], Lebanon [43], and Egypt [11], and known to carry carbapenemases [44], was encountered in 41.18% of the tested isolates harboring *bla*_OXA-48_ and/or *bla*_NDM_, which are among the carbapenemases frequently reported in Egypt [45]. Other ColRKp STs detected in our study: ST11, ST14, ST17, ST111, ST147, and ST525 have been witnessed disseminating globally [10, 46, 47]. Furthermore, O1 and O2 antigens accounted for 82.35% of the tested population, supporting the earlier finding that both serotypes are more frequently encountered in clinical *K. pneumoniae* isolates [48]. Therefore, genotyping of ColRKp isolates is essential to track the different clones and serotypes behind a threat endangering the global health situation.

Virulome analysis of the sequenced isolates highlighted the ubiquitous presence of capsular polysaccharide, LPS, *fimABCDEFGHIK*, and *mrkABCDFHIJ* gene clusters, which are essential pathogenicity factors for the early establishment of infection and bacterial evasion of host immune response [49]. The propensity of an infectious disease occurrence is further enhanced through the acquisition of yersiniabactin, aerobactin, and salmochelin siderophore systems [49] which were carried by ˃ 50% of the isolates, thus ensuring the thriving of *K. pneumoniae* isolates in iron deficient environment imposed by the host. Notably, *iuc*, which was carried by 52.94% of the isolates, is reported to play the utmost significant role in virulence among the four siderophore systems, both in vivo and in vitro [50]. Furthermore, *iuc, rmpA*, and *rmpA2* were detected in K7 isolate of ST383 lineage. ST383 is a non-hypervirulent clone [51] and its acquisition of hypervirulence biomarkers is a concerning scenario that might elicit increased morbidity and mortality, similar to the fatal outbreak witnessed in China due to a classical ST11 harboring these biomarkers [52]. Another important observation is the isolates tolerance for heavy metals, a phenomenon postulated to arm the isolates with enhanced survival capabilities in environmental habitats beyond hospital settings [53]. In response to this, proactive strategies and periodically updated infection control measures are imperative to prevent potential hospital outbreaks and subsequent community epidemics with highly virulent strains.

By inspecting the antibiotic resistance profiles displayed by ColRKp isolates, alarmingly high rates (70–100%) of resistance were detected towards almost all tested antibiotics except for TGC and DO which showed better efficacy against 88.24% and 52.94% of the collected XDR/MDR isolates, respectively, pointing them out as the available option for combating *K. pneumoniae* infections. The resistance profile pictured in this work could be correlated to an international surveillance study conducted in the ICUs of 88 countries which drew attention to the increased rates of AMR in ICUs compared to other hospital departments [54]. This could be ascribed to the unconstrained use of antibiotics in the ICUs for the urgency of initiating an empirical therapy, a practice adopted globally due to the inherent delay of conventional culture and susceptibility testing. Accordingly, up-to-date hospital antibiograms based on local epidemiological data are fundamental for guiding clinicians to select the most appropriate empirical treatment not only to ensure optimal patient outcomes but also to de-escalate the mounting risk of multidrug resistance.

The convergence of multiple antibiotic resistance and virulence genes in the same strain enhances its pathogenicity and complicates its treatment in an era where we are running out of antibiotics [55]. Although CZA has emerged over the last few years as a newly proposed antimicrobial agent to treat CRKP infections, it is still ineffective against NDM-producing CRKP [56]. Comparing the frequency of different detected carbapenemases, *bla*_NDM_ was found to prevail in 16/17 isolates, rendering CZA of a circumscribed clinical utility. The validity of our data was corroborated by a 1-year-cross-sectional study on Gram-negative bacteria conducted at Mansoura University Hospitals in 2019 [57] and a 2-year study on *Enterobacteriaceae* across 40 countries worldwide [58], wherein *bla*_NDM_ was the predominant gene correlated with carbapenem resistance.

Comparative analysis of phenotypic-genotypic antimicrobial resistance patterns (Fig. 2 and Additional file 2: Table S5) showed high concordance, especially for β-lactams, β-lactams combinations, fluoroquinolones, tetracyclines, folate pathway antagonist, and polymyxins. Some discrepancies were observed in macrolides and aminoglycosides phenotypic-genotypic profile, where a phenotypically resistant pattern with no corresponding resistance determinants could be justified by the existence of other resistance genes or mechanisms that have not been explored in the study, and a phenotypically susceptible pattern displayed in the presence of resistant determinants could be attributed to the harboring of silent resistance genes.

In terms of colistin resistome analysis, chromosomally encoded genes: *phoPQ*, *pmrAB*, *crrB*, and *mgrB* locus with its transcriptional regulator gene *kdgR*, and its genetic environment including *yobH*, *yebO*, *yobF*, and *cspC*, were analyzed *in silico* along with the investigation of the acquired plasmid-borne *mcr* genes. These genes were chosen based on being the most reported mechanisms worldwide in ColRKp [59]. Additional chromosomal genes including *pmrC*, *pmrD*, *arnBCADTEF* operon, and the global transcriptional regulator *ramA* that activates genes responsible for LPS synthesis and Lipid A modification were inspected as they have been suggested to confer a secondary colistin resistome [60].

The predominant mechanism mediating colistin resistance was found to be via the genetic modulation of *mgrB* locus that occurred in 82.35% of the collected isolates. Our results are in line with the numerous number of reports nominating *mgrB* mutation to be the most frequently occurring mechanism involved in colistin resistance [59]. MgrB protein functions as a negative feedback regulatory component of the PhoPQ 2-component system (2CS) [12]. It represses the formation of the active phosphorylated PhoP, which in turn decreases PhoPQ signal transduction to *arnBCADTEF* and *pmrCAB* operons, thus lowering L-Ara4N and pEtN production that relies on the activation of the former operons [12]. Accordingly, genetic alterations in *mgrB* result in the upregulation of PhoPQ and PmrAB 2CS that are connected through PmrD protein, thereby promoting the production of the LPS-modifying molecules and consequently conferring colistin resistance [61]. Insertional inactivation of *mgrB* locus was on the top tier of the detected *mgrB* alterations, with IS*Kpn14*, a member of IS1 family, being the most predominant in 6/8 isolates, while a member of IS5 family, IS*Kpn26*, inactivated *mgrB* in the other two isolates. Our data is consolidated by the existing literature that highlights *mgrB* insertional inactivation as the most common pathway of MgrB dysfunction [9]. However, the prevalent occurrence of IS*Kpn14* is incompatible with the preexisting studies stating that IS5 family elements cause the most frequent inactivation of *mgrB* [9]. This might be attributed to the different geographical regions in which the studies were conducted, hence, studies from Saudi Arabia and China were consistent with our findings [62, 63]. IS*Kpn14* transposed at nucleotide positions − 37, + 35, + 81, + 118, and + 119 with the first transposition being in the promoter region which is presumed to negatively impact the expression of the gene [12] and the remaining transpositions being within *mgrB* resulting in gene splitting and generation of malfunctioning MgrB [64]. While the transposition of IS*Kpn26* was at nucleotide position + 74 of *mgrB* and this position seems to be a hotspot for the insertion sequence as reported in earlier studies [64, 65]. Among other mutational events occurring in *mgrB* are the G37S deleterious mutation in MgrB and the deletion of ∼ 1.7 kb segment comprising the entire *mgrB* locus, *yebO*, *yobF*, and *cspC*, as well as a partial sequence of *yobH*. In a study conducted in Brazil in 2021, a similar colistin resistance mechanism was reported and was attributed to the deletion of ∼ 1.3 kb segment including *kdgR*, *yobH*, *mgrb*, and *yebO* [66]. Importantly, the two latter mutational and deletion events had been experimentally validated by a complementation assay using pACYC-*mgrB* plasmid containing wild-type *yobH*-*mgrB*-*yebO* in a study conducted by Cannatelli et al. where colistin sensitivity was restored [67]. Additionally, a mutation of guanine to adenine at position − 10 upstream *mgrB* is speculated to confer colistin resistance as the mutation occurred at a critical region where the ribosomal binding site is typically situated [68], thereby this might affect the binding efficiency of the ribosome to the mRNA and consequently impacts MgrB synthesis process.

Other molecular mechanisms of colistin resistance are the mutations occurring in genes of the regulatory PhoPQ and PmrAB 2CS activating the signaling pathways of the 2CS [12]. This activation will eventually lead to the overproduction of L-Ara4N and pEtN and the modification of LPS, rendering *K. pneumoniae* resistant to colistin [12]. In the current study, we detected novel and preexisting mutations linked to colistin resistance. To the best of our knowledge, L322Q and Q435H substitutions in PhoQ and T89P substitution in PmrB, are unprecedented deleterious mutations conferring colistin resistance as predicted by PROVEAN tool. Nonetheless, deleterious alterations in PmrB (T134P) and PmrA (G53V and D86E) had been reported by prior studies [69–71]. Other mutations in PmrC and across *arnBCADTEF* operon (Fig. 3) reflected the divergent mutation profiles displayed by ColRKp isolates as stated by earlier reports [63, 72], however, the contribution of these mutations to colistin resistance remains to be unclear and requires further investigation and validation.

CrrAB, an additional 2CS, was completely absent in isolates of ST14 and ST383 lineages. The absence of this system is not reported to be associated with colistin resistance [63] due to its variable presence in *K. pneumoniae* strains [73].

Collectively, our results imply that chromosomal mutations were the main driver for colistin resistance in tested isolates, whereas, the plasmid-encoded *mcr-1.1* gene, which is more readily transmissible, was harbored by a single isolate of ST525 clone. ST525 first emerged as a new high-risk clone in Hungary in 2006, causing multiple nosocomial outbreaks in distant places around the country until 2012 [74]. Since then, other reports on ST525 in Norway [46], Romania [75], Tunisia [76], and Iran [47] emerged, threatening the public health situation globally. This high-risk clone detected in our study exhibited an XDR profile along with a dual chromosomal and plasmid-mediated colistin resistance mechanism.

The *mcr-1.1* gene was carried on pEGY_KP9814_MCR1, a multi-replicon IncHI2/IncHI2A plasmid. The backbone of this multi-replicon plasmid was previously identified in *Klebsiella*-, *E. coli*-, and *Salmonella*-derived *mcr* plasmids worldwide [77, 78]. Strikingly, the notable resemblance of pEGY_KP9814_MCR1 in *K. pneumoniae* obtained from a clinical origin in the current study to those sourced from *E. coli* of food and animal origins in Egypt (pEGY1-MCR-1 [79] and pEGYMCR_IncHI2 [80]), reflects the circulating tendency of this plasmid for trafficking between different members of *Enterobacteriaceae* and among different sources for which the unconstrained colistin use as an antibiotic-fortified feed and growth promoter in animals could be attributed. Our findings are in agreement with previous studies reported from other parts of the world pointing to the zoonotic transmission of *mcr*-bearing plasmids to humans [81–83]. This provides direct evidence in support of One Health concept that emphasizes the transmission of drug resistance across the environment, humans, and animals [84]. Regarding this, enforcement legislation on the irrational use of colistin in the agriculture sector is of significant importance along with providing professional education and training on AMR in veterinary, food production, and agriculture sectors for the aim of curbing the dissemination of colistin resistance in the animal food chain. Moreover, the similarity noted between pEGY_KP9814_MCR1 and the plasmids recovered from clinical *K. pneumoniae* in Asia (pAN65-1 [85], pKP121-1-mcr [86], pKP14052-MCR-1 [87]) indicates the epidemic trait of this plasmid, disseminating colistin resistance between continents.

Prior sequence analysis of *mcr-1.1* environment showed that the gene is flanked by a composite transposon, two copies of IS*Apl1*, that promotes its transposition and mobilization between DNA molecules [88]. Across evolutionary changes, this composite transposon has lost one or both copies of IS*Apl1* which will probably limit its transposition and prompt transfixing of *mcr-1.1* cassette into the carrier plasmid facilitating its dissemination [89]. Therefore, the aforementioned data explains the variable presence of IS*Apl1* bracketing *mcr-1.1* either in the designated pEGY_KP9814_MCR1 plasmid or the previously published plasmids used for comparison. Overall, this is the first report for *mcr-1.1*-borne plasmid sourced from clinical *K. pneumoniae* belonging to ST525 high-risk clone.

The present study adds to the insufficiently available literature on genomic characterization of ColRKp in Egypt and emphasizes how genomics and WGS-based analysis are indispensable to gain better insights into the genetic basis of bacterial resistance to antimicrobial agents. Meanwhile, we acknowledge the limitations of the study. These include the small number of tested isolates which could serve as a preliminary step toward conducting a broader surveillance program within our local vicinity. The virulence profile was predicted based on the isolates genome without conducting phenotypic detection of virulence attributes. Additionally, the study focused on genetic characterization using WGS rather than transcriptomic analysis, therefore the expression levels of genes contributing to colistin resistance such as *pmrC*, *arnBCADTEF*, *ramA*, and genes encoding efflux systems (*kpnEF* and *acrAB*) were not measured.

## Conclusion

The current study unprecedently reports a snapshot of the genomic profile of ColRKp clinical isolates in the Egyptian ICUs based on WGS data. The emergence of pathogenic colistin-carbapenem-resistant *K. pneumoniae* foreshadows a menacing crisis as the clinical utility of the last-resort antibiotics is significantly diminishing. Here, we present the divergent molecular mechanisms involved in colistin resistance and, hence, provide crucial information to optimize patient care and contain the spread of these pathogens.

### Supplementary Information

**Additional file 1: Figure S1.** IMVC biochemical test results for the tested *K. pneumoniae* isolates; **a** Indole production test showing negative result for *K. pneumoniae* indicated by yellow color in the upper amyl alcohol layer and positive result for the quality control strain *E. coli* ATCC 8739 indicated by pink color in the upper amyl alcohol layer; **b** Methyl red test showing yellow negative result for *K. pneumoniae* and red positive result for *E. coli* ATCC 8739; **c** Voges–Proskaeur test showing dark red color for* K. pneumoniae* and brown color for *E. coli* indicating positive and negative results, respectively; **d** Simmon’s citrate utilization test showing respectively, blue and green colored slants for *K.* *pneumoniae* and *E. coli* ATCC 8739. **Figure S2.** **a** Catalase test results: L represents *Lactiplantibacillus plantarum* ATCC 8014 which was used as a negative control. *E. coli *ATCC 8739 was used as positive control and tested *K. pneumoniae* isolates showed positive catalase; **b** Positive and negative urease test results for *K. pneumoniae* and *E. coli *ATCC 8739 indicated by pink and yellow color, respectively; **c** Triple sugar iron agar test result of *K. pneumoniae* isolates showing fermentation of sugars evidenced by acid production turning slant and butt from red to yellow in addition to gas production seen as bubbles or cracking of agar. **Figure S3.** Representative results of phenotypic detection of colistin resistance and presumptive identification of *mcr-1 *using the modified rapid polymyxin Nordmann/Poirel (MPNP) test in *K. pneumoniae* isolates. The wells of column 1 represent sterility control by adding 50 µL of 0.85% NaCl, whereas the wells of columns 2 to 12 correspond to colistin sensitive (Col-S) *E. coli *ATCC 8739, colistin-resistant *E. coli* EC13655, and the tested *K. pneumoniae* clinical isolates K1 to K9, respectively. Colistin resistance is demonstrated by the growth of the isolates in the presence of colistin, indicated by a yellow color in wells B3-B12. The presence of acid metabolites in colistin/EDTA-containing RPNP presumes the absence of *mcr-1* (the color is yellow in wells D3-D12). The photograph was taken after incubation of the plate for 4 h.

**Additional file 2: Table S1.** Demographic data of collected colistin-resistant *K. pneumoniae* isolates. **Table S2.** MIC Distribution of colistin determined by broth microdilution. **Table S3.** Assembly statistics of the 17 colistin-resistant *K. pneumoniae* draft genomes. **Table S4.** Plasmid replicon types and genetic determinants of virulence, heavy metal resistance and antimicrobial resistance of the colistin-resistant *K. pneumoniae* isolates. **Table S5.** Phenotypic and genotypic profiles of  antimicrobial resistance for *K. pneumoniae* isolates.

## Data Availability

The WGS dataset generated during the current study was deposited at NCBI repository under BioProject number PRJNA917066 with samples accession numbers (SAMN32518852–SAMN32518868) which correspond to isolates from K1-K17 https://www.ncbi.nlm.nih.gov/search/all/?term=PRJNA917066. Mutated *mgrB* of the sequenced isolates can be found at NCBI with GenBank accession numbers (OR146624-OR146636). Plasmid designated pEGY_KP9814_MCR1 is deposited at NCBI with accession number: OQ215737 https://www.ncbi.nlm.nih.gov/nuccore/OQ215737.1.

## Abbreviations

CRKP: Carbapenem-resistant *K. pneumoniae*
LPS: Lipopolysaccharide
ColRKp: Colistin-resistant *K. pneumoniae*
*mcr*: Mobile colistin resistance gene
MPNP: Modified rapid polymyxin Nordmann/Poirel
Col-R: Colistin-resistant
Col-S: Colistin-sensitive
CLSI: Clinical Laboratory Standards Institute
BMD: Broth microdilution
MLST: Multilocus sequencing typing
AMR: Antimicrobial resistance
mini-BAL: Mini-bronchoalveolar lavage
ST: Sequence type
CPS: Capsule polysaccharide
XDR: Extensively drug-resistant
WT: Wild-type
ESBL: Extended-spectrum β-lactamase
PR: Promoter region
2CS: 2-Component system
