# Supplementary material for: Genomic characterization of colistin-resistant Klebsiella pneumoniae isolated from intensive care unit patients in Egypt
Source: Ann Clin Microbiol Antimicrob. 2023 Sep 9;22:82. doi: 10.1186/s12941-023-00632-9 (PMC10492301; doi:10.1186/s12941-023-00632-9)
Supplement: Supplementary file 1 — Additional file 1: Figure S1. IMVC biochemical test results for the tested K. pneumoniae isolates; a Indole production test showing negative result for K. pneumoniae indicated by yellow color in the upper amyl alcohol layer and positive result for the quality control strain E. coli ATCC 8739 indicated by pink color in the upper amyl alcohol layer; b Methyl red test showing yellow negative result for K. pneumoniae and red positive result for E. coli ATCC 8739; c Voges–Proskaeur test showing dark red color for K. pneumoniae and brown color for E. coli indicating positive and negative results, respectively; d Simmon’s citrate utilization test showing respectively, blue and green colored slants for K. pneumoniae and E. coli ATCC 8739. Figure S2. a Catalase test results: L represents Lactiplantibacillus plantarum ATCC 8014 which was used as a negative control. E. coli ATCC 8739 was used as positive control and tested K. pneumoniae isolates showed positive catalase; b Positive and negative urease test results for K. pneumoniae and E. coli ATCC 8739 indicated by pink and yellow color, respectively; c Triple sugar iron agar test result of K. pneumoniae isolates showing fermentation of sugars evidenced by acid production turning slant and butt from red to yellow in addition to gas production seen as bubbles or cracking of agar. Figure S3. Representative results of phenotypic detection of colistin resistance and presumptive identification of mcr-1 using the modified rapid polymyxin Nordmann/Poirel (MPNP) test in K. pneumoniae isolates. The wells of column 1 represent sterility control by adding 50 µL of 0.85% NaCl, whereas the wells of columns 2 to 12 correspond to colistin sensitive (Col-S) E. coli ATCC 8739, colistin-resistant E. coli EC13655, and the tested K. pneumoniae clinical isolates K1 to K9, respectively. Colistin resistance is demonstrated by the growth of the isolates in the presence of colistin, indicated by a yellow color in wells B3-B12. The presence of acid me [file 12941_2023_632_MOESM1_ESM.docx]

**Figure S1**

**
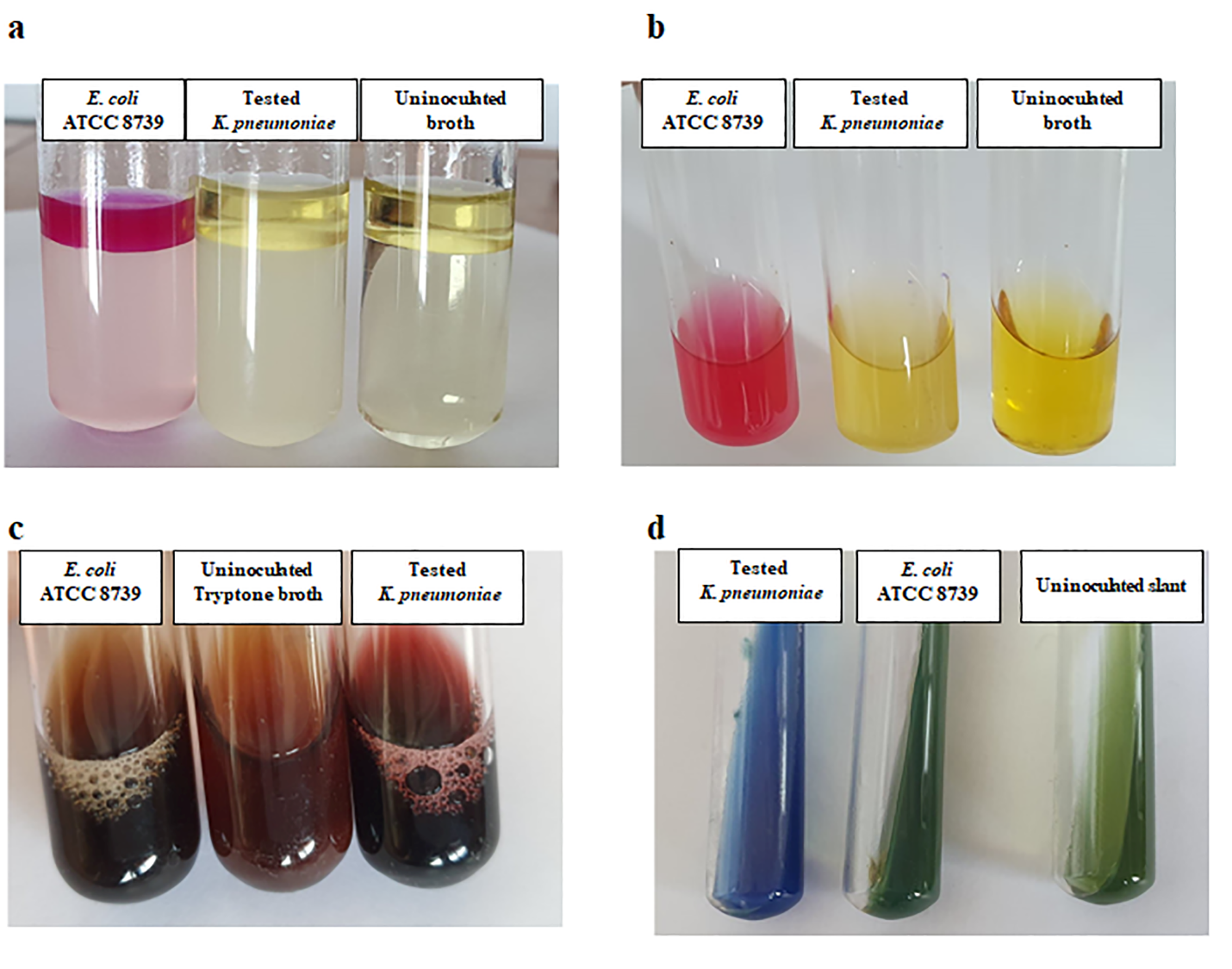
**

**Fig. S1** IMVC biochemical test results for the tested *K. pneumoniae* isolates; (**a)** Indole production test showing negative result for *K. pneumoniae* indicated by yellow color in the upper amyl alcohol layer and positive result for the quality control strain *E. coli* ATCC 8739 indicated by pink color in the upper amyl alcohol layer; **(b)** Methyl red test showing yellow negative result for *K. pneumoniae* and red positive result for *E. coli* ATCC 8739; **(c)** Voges-Proskaeur test showing dark red color for *K. pneumoniae* and brown color for *E. coli* indicating positive and negative results, respectively; **(d)** Simmon’s citrate utilization test showing respectively, blue and green colored slants for *K.* *pneumoniae* and *E. coli* ATCC 8739.

**Figure S2**

**
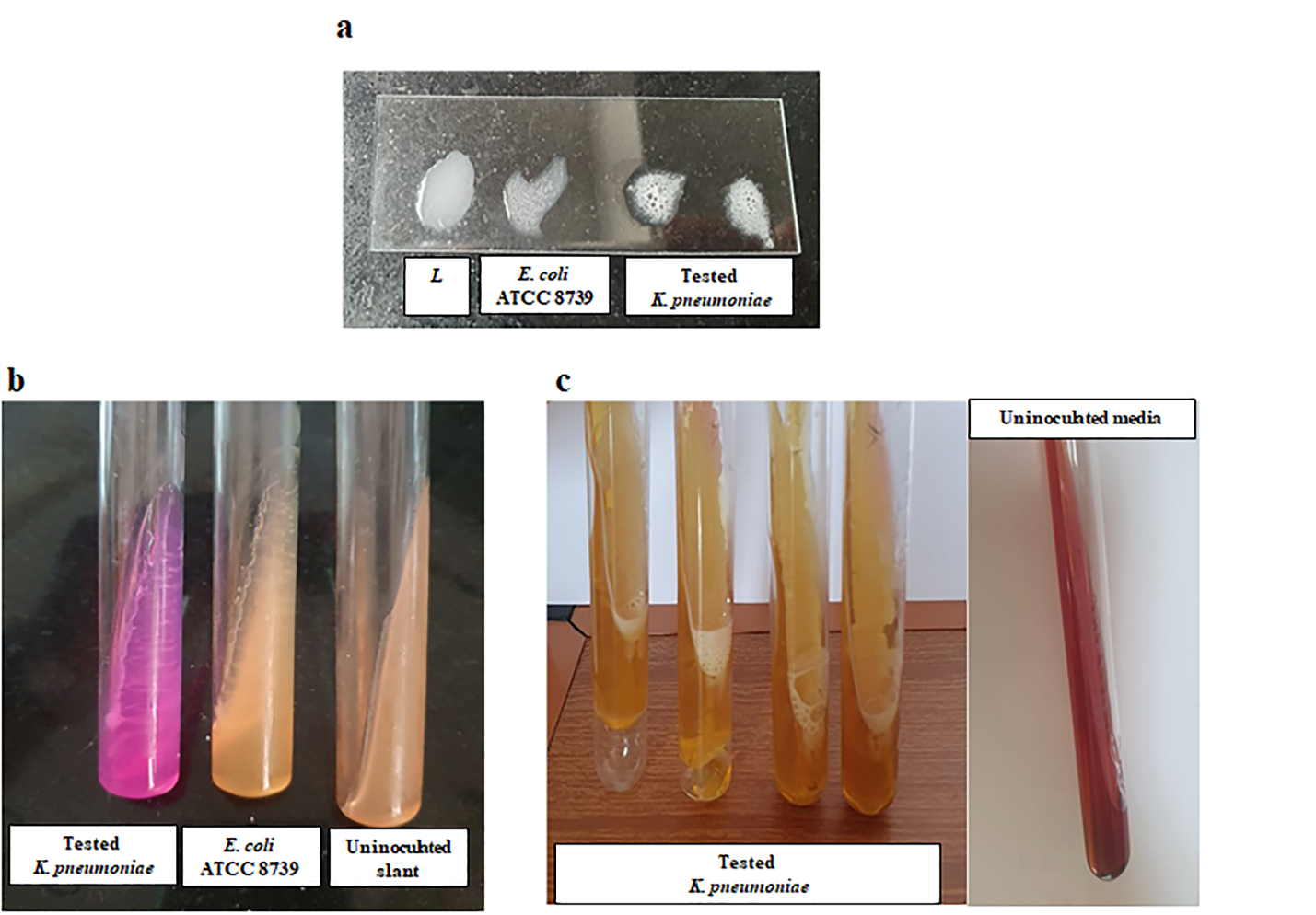
**

**Fig. S2 (a)** Catalase test results: L represents *Lactiplantibacillus plantarum* ATCC 8014 which was used as a negative control. *E. coli* ATCC 8739 was used as positive control and tested *K. pneumoniae* isolates showed positive catalase; **(b)** Positive and negative urease test results for *K. pneumoniae* and *E. coli* ATCC 8739 indicated by pink and yellow color, respectively; **(c)** Triple sugar iron agar test result of *K. pneumoniae* isolates showing fermentation of sugars evidenced by acid production turning slant and butt from red to yellow in addition to gas production seen as bubbles or cracking of agar.

**Figure S3**

**
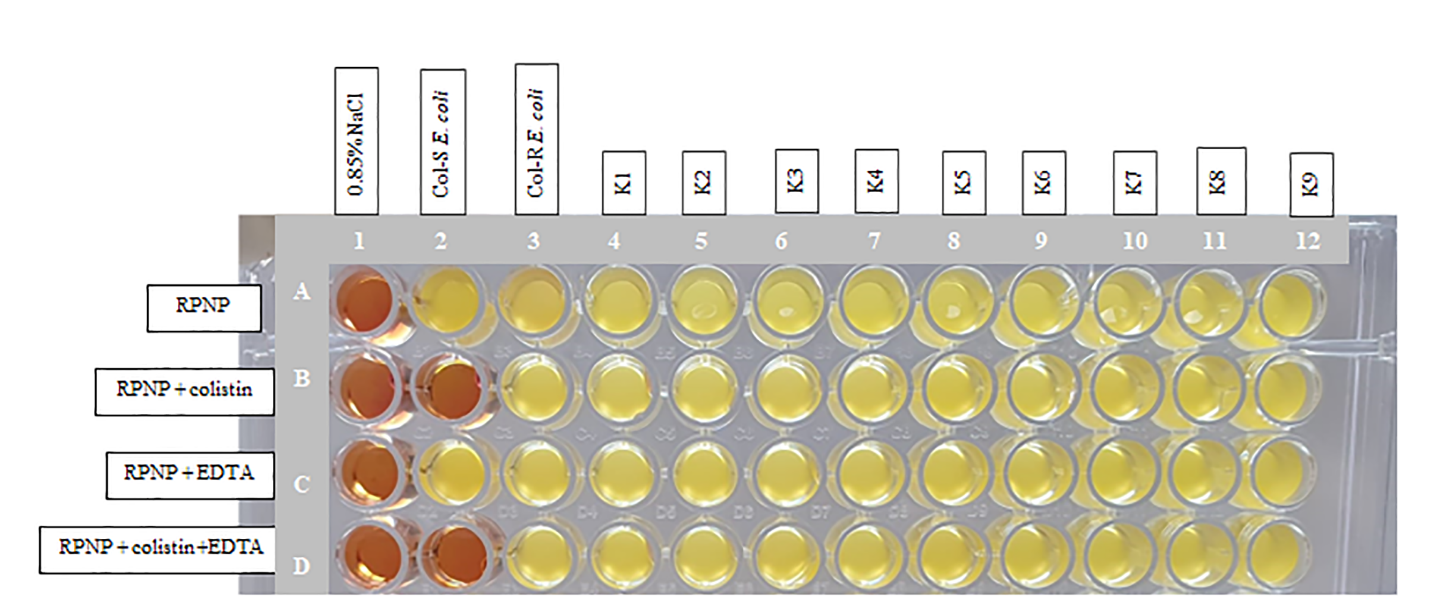
Fig.S3** Representative results of phenotypic detection of colistin resistance and presumptive identification of *mcr-1* using the modified rapid polymyxin Nordmann/Poirel (MPNP) test in *K. pneumoniae* isolates. The wells of column 1 represent sterility control by adding 50 µl of 0.85% NaCl, whereas the wells of columns 2 to 12 correspond to colistin sensitive (Col-S) *E. coli* ATCC 8739, colistin-resistant *E. coli* EC13655, and the tested *K. pneumoniae* clinical isolates K1 to K9, respectively. Colistin resistance is demonstrated by the growth of the isolates in the presence of colistin, indicated by a yellow color in wells B3-B12. The presence of acid metabolites in colistin/EDTA-containing RPNP presumes the absence of *mcr-1* (the color is yellow in wells D3-D12). The photograph was taken after incubation of the plate for 4 h.
